# Progressive familial intrahepatic cholestasis—outcome and time to transplant after biliary diversion according to genetic subtypes

**DOI:** 10.3389/fsurg.2023.1074229

**Published:** 2023-06-08

**Authors:** Abdulla Sahloul, Elke Lainka, Simone Kathemann, Sandra Swoboda, Carola Dröge, Verena Keitel, Yahya Saleh Al-Matary, Michael Berger, Maren Schulze

**Affiliations:** ^1^ Department of General, Visceral and Transplant Surgery, University Hospital Essen, University Duisburg-Essen, Essen, Germany; ^2^ Department of Pediatric Gastroenterology, University Hospital Essen, University Duisburg-Essen, Essen, Germany; ^3^ Department of Gastroenterology, Hepatology, and Infectious Diseases, University Hospital, Medical Faculty, Heinrich Heine University Düsseldorf, Düsseldorf, Germany; ^4^ Department of Gastroenterology, Hepatology, and Infectious Diseases, Medical Faculty, Otto von Guericke University Magdeburg, Magdeburg, Germany; ^5^ Department of Dermatology, University Hospital Essen, Essen, Germany; ^6^ King Faisal Specialist Hospital & Research Centre, Organ Transplant Centre of Excellence, Riyadh, Saudi Arabia

**Keywords:** biliary diversion, morbus byler, pediatric liver transplantation (pediatric LT), progressive familial intrahepatic cholestasis (PFIC), pediatric surgery, liver disease

## Abstract

**Background:**

Progressive familial intrahepatic cholestasis (PFIC) is a heterogeneous disease characterized by progressive cholestasis in early childhood. Surgical therapy aims at preventing bile absorption either by external or internal biliary diversion (BD). Several different genetic subtypes encode for defects in bile transport proteins, and new subtypes are being discovered ongoingly. Overall, the literature is scarce, however, accumulating evidence points to PFIC 2 having a more aggressive course and to respond less favorable to BD. With this knowledge, we aimed to retrospectively analyze the long-term outcome of PFIC 2 compared to PFIC 1 following BD in children at our center.

**Methods:**

Clinical data and laboratory findings of all children with PFIC, who were treated and managed in our hospital between 1993 and 2022, were analyzed retrospectively.

**Results:**

Overall, we treated 40 children with PFIC 1 (*n* = 10), PFIC 2 (*n* = 20) and PFIC 3 (*n* = 10). Biliary diversion was performed in 13 children (PFIC 1, *n* = 6 and 2, *n* = 7). Following BD, bile acids (BA) (p = 0.0002), cholesterol (p < 0.0001) and triglyceride (p < 0.0001) levels significantly decreased only in children with PFIC 1 but not in PFIC 2. Three out of 6 children (50%) with PFIC 1 and 4 out of 7 children (57%) with PFIC 2 required liver transplantation despite undergoing BD. On an individual case basis, BA reduction following BD predicted this outcome. Of the 10 children who had PFIC 3, none had biliary diversion and 7 (70%) required liver transplantation.

**Conclusion:**

In our cohort, biliary diversion was effective in decreasing bile acids, cholesterol levels as well as triglycerides in the serum only in children with PFIC 1 but not PFIC 2. On an individual case level, a decrease in BA following BD predicted the need for liver transplantation.

## Introduction

Progressive familial intrahepatic cholestasis (PFIC) constitutes a group of rare cholestatic disorders. There are several different subtypes based on mutations of hepatocellular transport system genes involved in the formation of bile, and recently new subtypes have been described (1). Depending on the genetic subtypes, clinical presentation can vary significantly but typically involves recurrent cholestasis, pruritus and in some cases deterioration of liver function, which can lead to cirrhosis and end stage liver disease.

Biliary diversion, either as partial external biliary diversion (PEBD) or internal diversion, is known to sometimes slow liver damage and prevent or at least delay the need for liver transplant, at least for some genetic subtypes, such as PFIC 1 (2). In comparison, children with PFIC 3 slowly progress to liver fibrosis and cirrhosis, and typically these children present late in their course in need for liver transplant evaluation.

Among the various genetic subtypes, mutations in the ABCB11 gene can lead to a deficiency of the bile salt export pump (BSEP), leading to intrahepatic cholestasis. The most severe form of BSEP deficiency has been labelled PFIC type 2 (3). PFIC 2 has recently been described to respond less favorable to surgical BD (2).

With this accumulating evidence, we aimed to analyze the long-term outcome of PFIC 1 and 2 following BD in children at our center over the last two decades.

## Patients and methods

### Patient cohort

For all children with PFIC treated at our hospital between 1993 and 2022, we collected clinical and social data, time of presentation, treatment, and time to transplant with or without biliary diversion procedures. Blood tests were assessed before the intervention, postoperatively, before and after transplantation until the last follow up. Some patients were transferred to our hospital specifically for elective biliary diversion or for evaluation of liver transplantation after having been diagnosed and treated elsewhere.

Liver biopsy was done in all patients, however, at very different time points. Initial therapy was conservative with fat soluble vitamins, Ursodeoxycholic acid (UDCA), phenobarbital, naltrexon, rifampicin and cholestyramine. All patients had regular 3 to 6 month follow ups. Those children without signs of advanced fibrosis or cirrhosis on liver histology and persistent pruritus despite medical therapy were evaluated for BD.

### Biliary diversion

**Figure 1:**
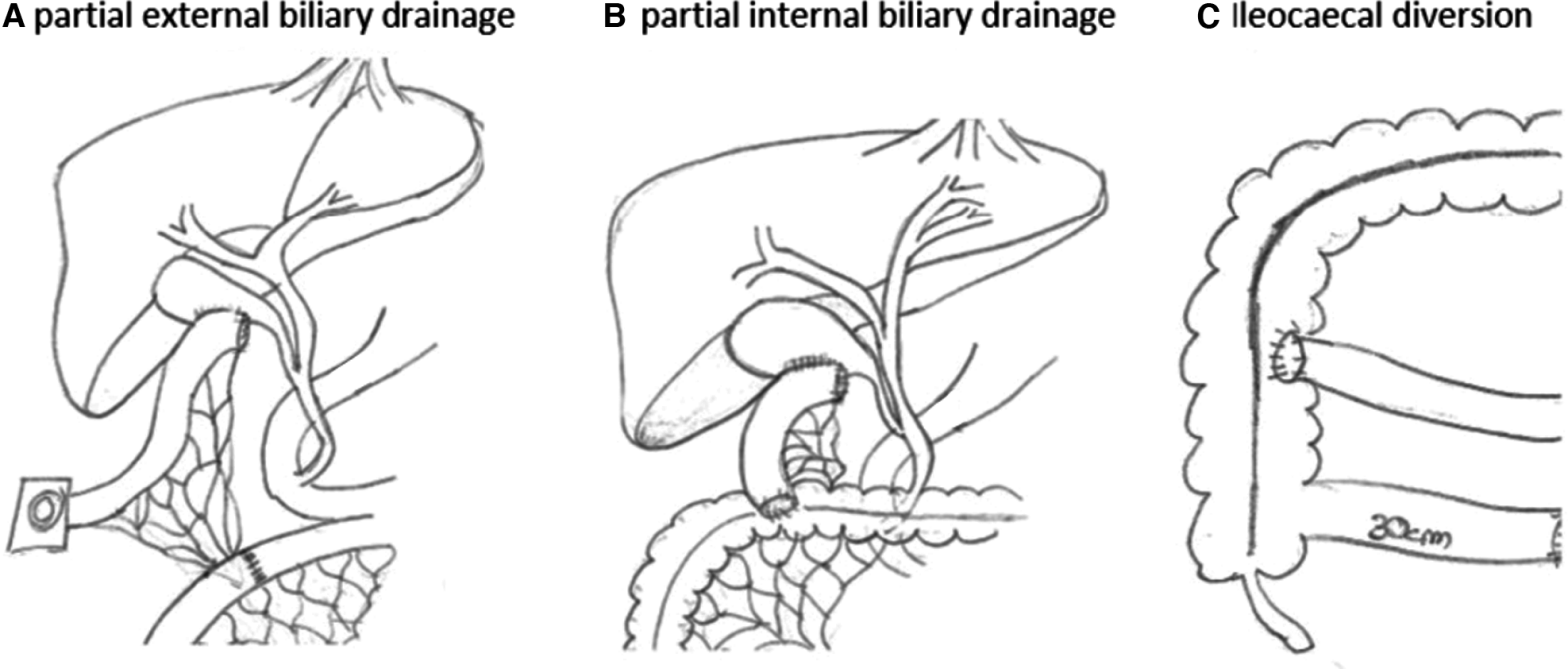
Partial external or internal biliary diversion. In partial external biliary diversion (**A**) a small bowel segment is interpositioned between the gallbladder and the skin and diverted as a stoma. Alternatively, the gallbladder can be diverted directly to the skin. Partial internal biliary diversion (**B**) drains bile from the gallbladder via an interpositioned loop directly into the colon. Ileocaecal diversion (**C**) reduces the amount of bile reabsorbed in the terminal ileum. Drawings by the author (M.S.).

The indication for biliary diversion was considered individually. The indication for biliary diversion was made for children who did not yet have advanced fibrosis or liver cirrhosis and who, despite drug therapy, showed a deterioration in liver status or persistent itching. All children were subjected to partial external biliary diversion as our default surgical option. In this procedure, the gallbladder is either directly diverted as a stoma or with an interposition of a small bowel segment (Figure 1). The operation can safely be carried out either laparoscopically or open. Variations of this procedure were carried out on an individual case basis and are explained throughout the text when appropriate.

### Laboratory collection

Documented laboratory values were collected for all patients, regardless of the period in which they were taken, and initially assessed for all patients prior to the intervention. The laboratory values were also collected, evaluated, and analyzed postoperatively until the liver transplantation or the last appearance of the patients. When collecting data after BD-operation, laboratory values were collected directly, at one week, at discharge, at three months and at six months. To compare pre- and postoperative laboratory values, the corresponding values were pooled to generate a mean. This was necessary due to the large number of blood samples analyzed per patient. Due to the long study period of two decades and the consideration of samples collected at other hospitals, patients typically accumulated dozens of samples, with one patient peeking at 135 blood samples.

### Statistics

Results are expressed as the mean ± standard error of the mean (SEM). All statistical comparisons were made with an unpaired parametric *t*-test comparing two groups, anordinary one-way ANOVA using GraphPad Prism (San Diego, CA, USA). The significance was considered as: *p* < 0.05 (*), *p* < 0.01 (**), *p* < 0.001 (***) and *p* < 0.0001 (****) for all comparisons. To perform Kaplan-Meier analysis in R, we used the survfit() function from the survival package. The survdiff() function was used to run the log-rank test, with the corresponding *p*-values shown in the graph (significance of the test was considered if the *p*-value was less than *α *= 0.5 (95% confidence intervall).

## Results

### Biometric and clinical data

Between 1993 and 2022, we treated 40 patients with PFIC. Most common was PFIC 2 with 20 cases followed by PFIC 1 and PFIC 3 with 10 cases each. Twenty-one patients were male, 19 were female. Biliary diversion was performed in 13 children (6 with PFIC 1 and 7 with PFIC 2). Most common symptoms were jaundice, coagulopathy in PFIC 2 and pruritus in PFIC 1. Two patients with PFIC 2 had previously been diagnosed with liver tumors at time of presentation, which were proven to be HCC after histological examination. Clinical course in children with PFIC 3 was not as severe presenting with mild pruritus and slow progression of liver disease, most of them presenting late for liver transplant evaluation. Therefore, in our cohort, PFIC 2 was most frequent (Table 1). Average age of the patients at presentation was 2.86 years with a range from one month to 13 years (Table 1). Some patients presented late in the course of their disease. With an average age of 1.7 months (range 0–3 months), the clinical symptoms in PFIC 2 appeared early in neonatal age and/or in infancy while in PFIC 1 with a mean age of 11.7 months (range 3–23 months) somewhat later.

**Table 1:** Patient values at baseline.

	Total	PFIC 1	PFIC 2	PFIC 3
	*n* = 40	*n* = 10	*n* = 20	*n* = 10
	Mean (SD) or %	Mean (SD) or %	Mean (SD) or %	Mean (SD) or %
Gender				
Male, *n* (%)	21 (52)	4 (40)	13 (65)	4 (40)
Female, *n* (%)	19 (48)	6 (60)	7 (35)	6 (60)
Baseline characteristics				
Age at presentation (years)	2.86 (± 4.29)	3.38 (± 5.10)	2.50 (± 3.50)	3.25 (± 6.5)
Age at BD-operation (years)	4.31 (± 4.92)	4.33 (± 4.32)	4.29 (± 5.74)	
Age at LTx (years)	7.08 (± 5.92)	6.6 (± 7.64)	4.62 (± 4.61)	12 (± 4.12)
Time from BD to LTx (month)	54.88 (± 53.99)	94 (± 66.51)	39.25 (± 31.49)	
Laboratory values				
Bile acids (µmol/L)	232.91 (± 158.81)	222.06 (± 111.39)	274.69 (± 180.41)	166.39 (± 135.81)
Total Bilirubin (mg/dl)	6.81 (± 7.37)	6.05 (± 3.53)	7.99 (± 8.45)	5.2 (± 7,89)
Direct Bilirubin (mg/dl)	5.45 (± 6.12)	4.67 (± 3.30)	6.45 (± 7.08)	4.18 (± 6.15)
AST (U/L)	175.35 (± 138.76)	106.92 (± 75.50)	240.39 (± 163.76)	113.69 (± 47.88)
ALT (U/L)	117.25 (± 83.22)	86.27 (± 61.53)	152.52 (± 95.42)	77.7 (± 37.83)
gGT (U/L)	104.37 (± 238.00)	17.41 (± 6.84)	34.2 (± 17.37)	331.64 (± 409.92)
Alkaline Phosphatase (U/L)	593.93 (± 464.23)	969.21 (± 734.44)	507.78 (± 257.3)	382.33 (± 127.36)
Cholesterol (mg/dl)	167.58 (± 63.32)	182.20 (± 72.94)	149.43 (± 47.8)	200.75 (± 71.51)
Triglycerides (mg/dl)	187.12 (± 120.43)	234.28 (± 79.96)	176.25 (± 82.44)	165.32 (± 193.24)

The child's nationality was documented in 15 cases. Six families were from Turkey, three from Hungary, one from Iraq, two from Russia, one from Slowakia and two from Germany. Of note, for 10 parents there was some form of consanguinity documented.

If an indication for biliary diversion was present, all children underwent PEBD except one patient who received internal biliary diversion because a cholecystectomy had previously been performed. For children undergoing PEBD, high output stoma, electrolyte depletion, stoma obstruction, as well as stoma-leakage and recurrent cholangitis were documented complications occurring after biliary diversion (high-output stoma developed in two children). One girl with PFIC 1 had a PEBD performed at age 3 years and was converted to an internal drainage at age 11 years due to the family's wishes. She has not required liver transplant in the 5-year follow-up period since. One boy with PFIC 1 underwent PEBD at another hospital at age 6 years and initially progressed well. Due to the family's and the boys wishes, at age 16 years, he underwent a stoma takedown and button-placement into the conduit for continued external biliary drainage. Following this procedure, significant stoma problems developed, and the patient underwent conversion to internal drainage 3 years later. He suffered a bile leakage requiring subsequent emergent laparotomy in the postoperative period. Since this last procedure, his liver function deteriorated, and he was finally transplanted at age 19 years. One male child with PFIC 2 had a PEBD at age 1 year and progressed well subsequently. His bile acids and direct bilirubin levels where high preoperatively (252.7 µmol/L and 6.2 mg/dl, respectively) and both nicely fell below the reference value. He had his stoma taken down 10 years later due to the family's wishes and, 1 year following the takedown, has not yet had new symptoms or a rise in bile acids or direct bilirubin levels.

### Laboratory values before the intervention

**Figure 2:**
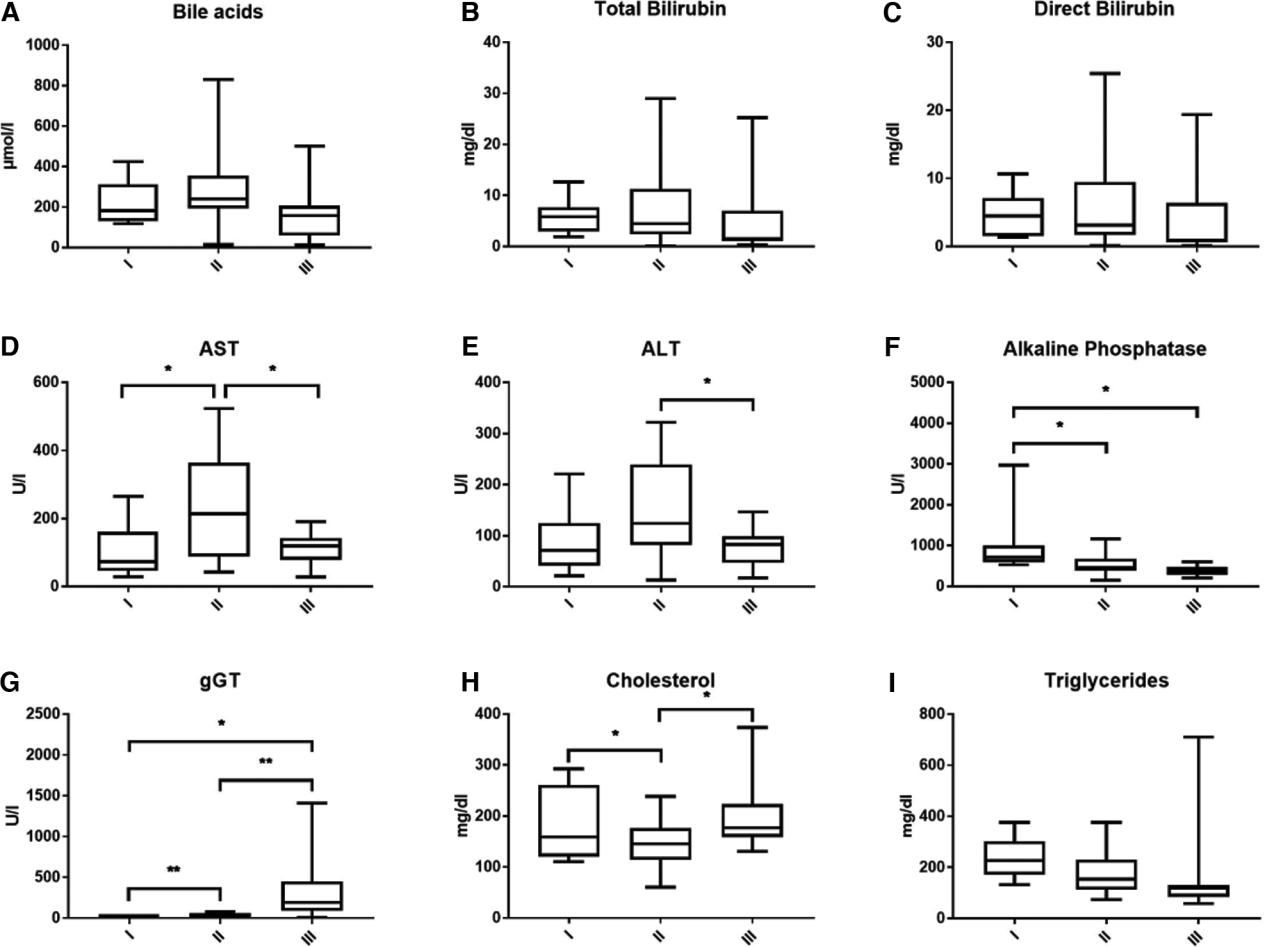
This figure shows the average bile acids (**A**), total and direct bilirubin (**B,C**), transaminases (**D,E**), cholestatic parameters (**F,G**) and lipid values (**H,I**) according to subtypes at time of presentation.

Bile acid levels were consistently high in all children with PFIC and highest in PFIC 2 (Figure 2A). Mean bile acid was 222.06 ± 111.39 (range 117.95–424.5 µmol/L) in PFIC 1, 274.69 ± 180.41 (range 14.84–829.65 µmol/L) in PFIC 2 and 166.39 ± 135.81 (range 12.86–500.67 µmol/L) in patients with PFIC 3, respectively. Bilirubin levels were elevated without significant difference between the groups (Figures 2B,C). Total bilirubin was higher in children with PFIC 2 (mean 7.99 ± 8.45, range 0.1–29.02 mg/dl) compared to the other two groups [PFIC 1 6.05 ± 3.53 (range 1.92–12.6 mg/dl) and PFIC 3 5.2 ± 7.89 (range 0.35–25.26 mg/dl)]. Direct bilirubin levels were of 4.67 ± 3.30 (range 1.4–10.66 mg/dl) for PFIC 1, of 6.45 ± 7.08 (range 0.1–25.42 mg/dl) for PFIC 2 and of 4.18 ± 6.15 (range 0.13–19.39 mg/dl) for PFIC 3. The elevation of bilirubin in children with PFIC 2 was, however, not statistically significant.

AST and ALT were elevated in all PFIC patient groups. AST was significantly higher in PFIC 2 with an average value of 240.39 ± 163.76, p = 0.0215, (range 42.68–523.43 U/L) than in PFIC 1 106.92 ± 75.5 (range 28.3–264.83 U/L) and in PFIC 3 with a mean of 113.69 ± 47.88 (range 27.92–190.67 U/L). Mean ALT in children with PFIC 2 was 152.52 ± 95.42 (range 13–321.67 U/L), higher than in PFIC 1 with a mean of 86.27 ± 61.53 (range 21–221 U/L) and significantly higher (*p* = 0.0249) than in PFIC 3 (mean 77.7 ± 37.83 (range 17–146.64 U/L) (Figures 2D,E).

Alkaline phosphatase was increased in all PFIC patient groups, with an average of 969.21 ± 734.44 (range 527–1,220.25 U/L) in PFIC 1, and significantly higher (*p* = 0.019) than in children with PFIC 2 with an average of 507.78 ± 257.3 (range 145.11–857.93 U/L) and children with PFIC 3 with an average of 382.33 ± 127.36 (range 201–592.06 U/L, p = 0.0228) (Figure 2F). gGT was significantly higher in children with PFIC 3 than in PFIC 1 and PFIC 2 (Figure 2G). There was a significant difference for cholesterol between PFIC 2 and both other groups (Figure 2H). Mean values of the triglycerides in PFIC 3 with an average of 165.32 ± 193.24 (range 58 –710 mg/dl) were lower than in PFIC 1 (234.28 ± 79.96, range 133–327.8 mg/dl), and PFIC 2 (176.25 ± 82.44, range 74.5–301.2 mg/dl).

### Development of laboratory values after biliary diversion

**Figure 3:**
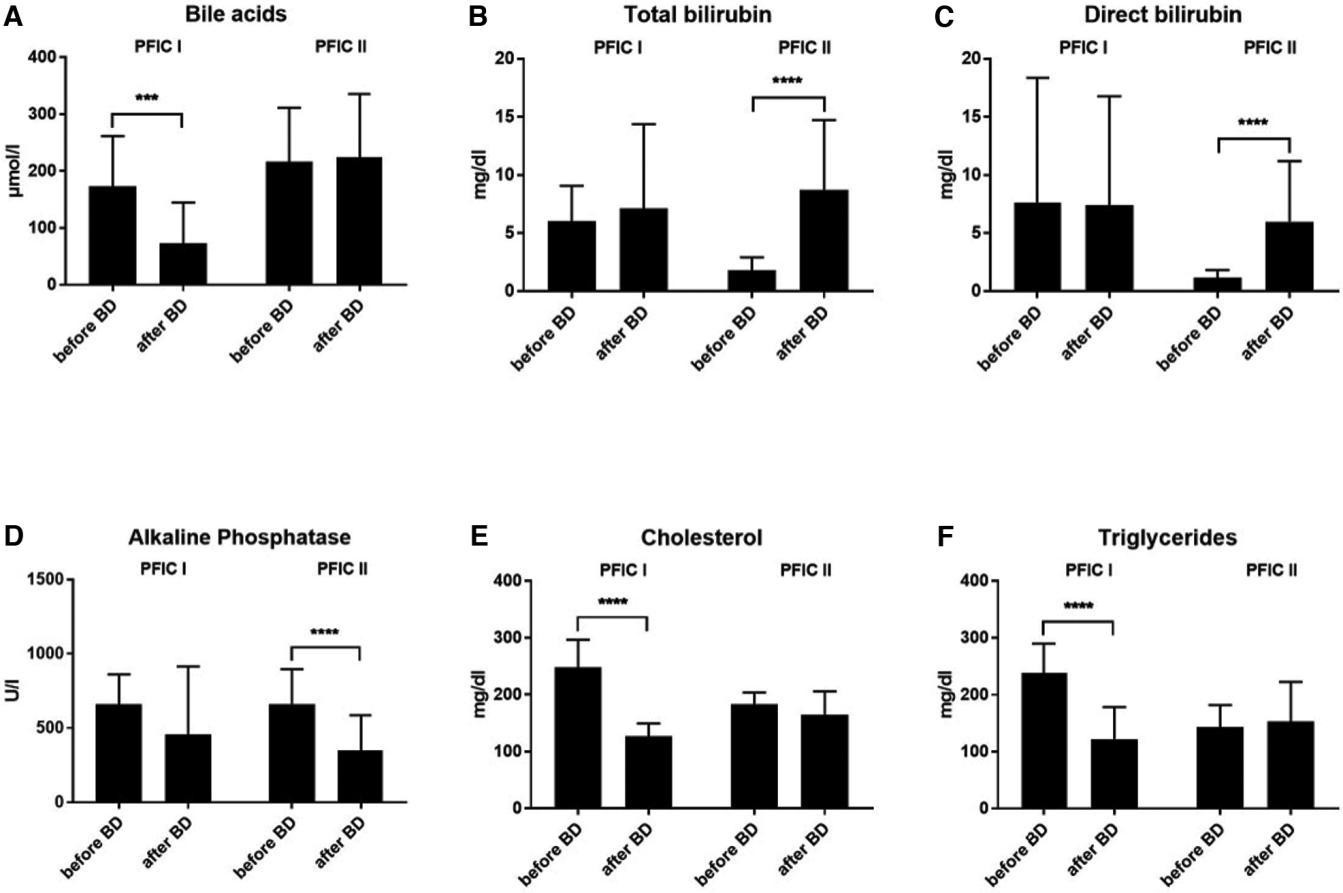
The development of laboratory values after biliary diversion in both PFIC 1 and 2. (**A**) bile acids, (**B**) total bilirubin, (**C**) direct bilirubin, (**D**) alkaline phosphatase and (**E,F**) lipid values.

Biliary diversion was performed in 13 children with PFIC 1 (*n* = 6) and 2 (*n* = 7). After biliary diversion, children with PFIC 1 experienced a dramatic decrease in bile acid levels from 173.4 ± 88.19 (range 59.6–261 µmol/L) preoperatively to 72.84 ± 71.66 (range 0.4–308.7 µmol/L) postoperatively, p = 0.0002 (Figure 3A). Subsequently, there was both an improvement of liver function and clinical symptoms. Interestingly, when pooled in our cohort the bile acid values of the children with PFIC 2 remained without change after the biliary diversion. The bile acid values for PFIC 2 were 216.1 ± 95 (range 8.6–528.0 µmol/L) preoperatively and 223.7 ± 111.7 (range 2.1–596.4 µmol/L) postoperatively (Figure 3A).

In a similar sense, the concentration of bilirubin in children with PFIC 2 increased after biliary diversion, whereas there was no change in children with PFIC 1 (Figures 3B,C). The total bilirubin values in children with PFIC 2 increased from 1.77 ± 1.16 (range 0.1–8.5 mg/dl) preoperatively to 8.75 ± 5.98 (range 0.1–24.0 mg/dl) postoperatively *p* < 0.0001, the values of direct bilirubin increased from 1.13 ± 0.68 (range 0.1–7.7 mg/dl) preoperatively to 5.97 ± 5.21 (range 0.1–19.7 mg/dl). On the other hand, there was no improvement in the postoperative values of either total bilirubin [7.12 ± 7.25 (range 0.2–31.2 mg/dl)] nor direct bilirubin [7.42 ± 9.36 (range 0.1–28.6 mg/dl)] in PFIC 1. The preoperative value of total bilirubin was 6.04 ± 3.03 (range 1–18 mg/dl) and of direct bilirubin was 7.64 ± 10.72 (range 0.6–44 mg/dl) (Figures 3B,C).

The alkaline phosphatase values did not normalize after biliary diversion in either group (PFIC 1 and 2, Figure 3D). Despite higher postoperative values, the AP dropped significantly in children with PFIC 2 from 661.7 ± 235.2 (range 228– 1073 U/L) preoperatively to 347.9 ± 238.2 (range 107–7497 U/L) postoperatively, *p* < 0.0001.

The mean values for cholesterol were high preoperatively in both groups. Postoperatively the cholesterol levels normalized only in children with PFIC 1, with *p* < 0.0001. The mean value for cholesterol in children with PFIC 1 was 247.9 ± 48.45 (range 129–307 mg/dl) preoperatively and fell to 126.6 ± 22.56 (range 92–179 mg/dl) postoperatively, while the cholesterol mean value in children with PFIC 2 was 182.6 ± 21.23 (range 144–237 mg/dl) and remained similar postoperatively (Figure 3E).

The triglyceride mean values in both groups were high preoperatively and normalized after biliary diversion only in children with PFIC 1, whereas no decrease was discernible in children with PFIC 2 (Figure 3F). The triglyceride mean in children with PFIC 1 fell from 238 ± 51.71 (range 135–376 mg/dl) preoperatively to 122.2 ± 56.39 (range 39–293 mg/dl) postoperatively, *p* < 0.0001. The postoperative mean value of triglyceride in children with PFIC 2 of 152.9 ± 69.48 (range 64–376 mg/dl) remained within the postoperative range of 142.9 ± 38.83 (range 85.14–364.5 mg/dl).

### Native liver survival

Of the 10 children who had PFIC 1, 5 (50%) had liver transplantation. Six (60%) of these children had undergone biliary diversion, and 3 children in that group needed liver transplantation. One child had a closure of the stoma as a teenager due to psychological problems. However, liver function deteriorated, and liver transplantation became necessary due to liver dysfunction and increasing cholestasis. Liver transplantation was needed in 50% of children with PFIC 1 who had not received biliary diversion.

**Figure 4:**
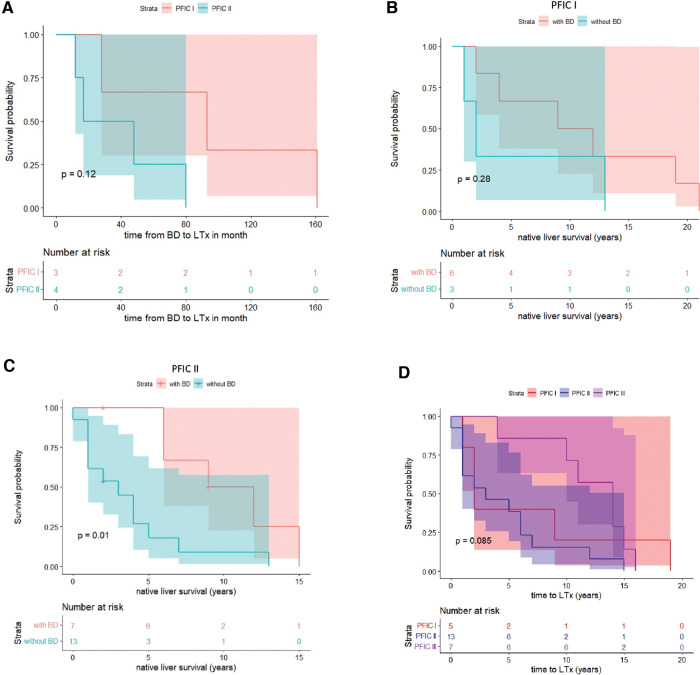
Kaplan-Meier survival curve of PFIC 1 vs. PFIC 2 subgroups for time from BD to LTx (**A**) and native liver survival (**B–D**).

Of the 20 children who had PFIC 2, 13 (65%) had liver transplantation. Seven (35%) of these children had undergone biliary diversion, and liver transplantation was required in 4 (57%) of children with PFIC 2 after the biliary diversion. One patient died before the liver transplant. Overall, liver transplantation was performed in 5 children with PFIC 1 (average time: 6.6 years) and 13 children with PFIC 2 (average time: 4.62 years). The period between BD and LTx was significantly longer in children with PFIC 1 than in PFIC 2 (Table 1 and Figure 4A).

### Bile acid levels following BD predict need for liver transplantation

**Figure 5:**
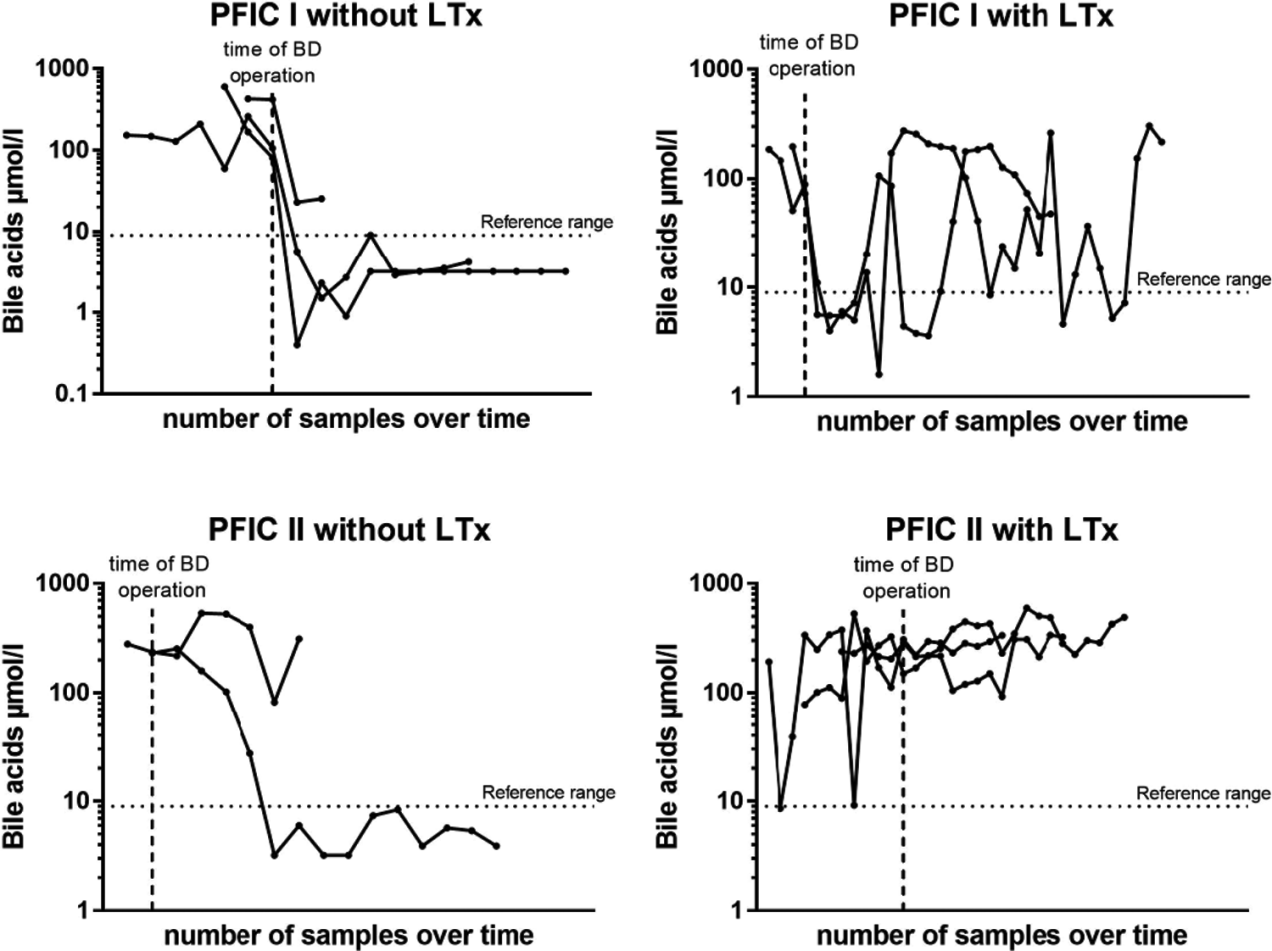
Bile salts levels on an individual case bases in relation to the time point of BD for PFIC subtype and liver transplantation. Each dot represents one blood sample analysis, allowing for appreciation of the evolution of each case over time.

In another step, we analyzed the bile acid levels on a case per case basis over time and in relation to BD. For those 10 children, in which both pre- and postoperative bile acid levels were available for comparison and who underwent BD, we found that a drop of bile acid levels below the age-adjusted reference range strongly correlated with native liver survival, independently of the subtype. Rather, of the 7 children whose bile acids levels did not reliably and persistently drop below the reference range, 5 (71,4%) went on to undergo liver transplant. On the other hand, of the remaining 3 children whose bile acid levels did drop below the reference range and stayed there following BD, none required liver transplantation (Figure 5).

## Discussion

In this study, we reviewed the surgical outcome of children with PFIC subtypes 1 and 2 over a 19-year period at a single university hospital in Germany. Our findings demonstrated that only children with a reduction of bile acids after biliary diversion benefited from the operation in the sense that native liver survival was prolonged. These were mainly children with PFIC 1. In this collective liver transplantation could only be avoided or significantly delayed if bile acids normalized after biliary diversion (Figure 5). On the contrary, children with PFIC 2 showed poor response to surgical BD. Either way, a drop of bile acid levels below the age-adjusted reference range following surgical BD strongly correlated with native liver survival, independently of the genetic subtype.

The prominent difference in clinical behavior following biliary diversion between PFIC 1 and 2 described in our study is in accordance with data of others (2). It is likely explained by the fact that genes encoding for PFIC 1 and 3 are located at the canalicular membrane for bile salt absorption, whereas the gene defect for PFIC 2 BSEP (ABCB11) is for bile excretion. Therefore, bile diversion does not interfere with the pathomechanism of PFIC 2, and accumulation of cytotoxic bile acids within the hepatocyte is not altered by biliary diversion. Lemoine et al. in 2017 (2) published a series of 24 children who underwent BD for PFIC. In their cohort, 10 children had BD for PFIC 1, 13 for PFIC 2 and one for PFIC 3. Like our study, following BD, BA levels decreased in children with PFIC 1, but not significantly in children for PFIC 2. Five-year transplant-free survival was 100% in children for PFIC 1, but only 38% in children for PFIC 2. In this same sense, regarding patients with PFIC 2, van Wessel et al. recently analyzed a total of 264 patients with homozygous or compound heterozygous pathological ABCB11 mutations (3). Patients were categorized according to genotypic severity (BSEP1, BSEP2, BSEP3) in which the predicted residual BSEP transport function decreased with each category. Importantly, in their analysis they found that for this high-risk cohort (PFIC 2) genotype severity was strikingly associated with native liver survival (median NLS of 20.4 years for BSEP1 vs. 3.5 years for BSEP3; *p* < 0.001). Interestingly, and similar to our results, within this high-risk group of children with PFIC 2, surgical BD was associated with significantly increased NLS when a significant drop in serum bile acid concentration (of more than 75%) following surgical BD was observed. In those cases, a drop in BA acids reliably predicted NLS. Interestingly, this effect was observed only when analyzing patients with BSEP1 and BSEP2. The effect was lost when patients with BSEP3 were included in the analysis.

Biliary diversion has long been considered the gold standard for children with severe pruritus in PFIC or Alagille syndrome in the setting of otherwise good liver function (4). The evolution of this procedure from its initials description in the 1980s was recently reviewed in detail by Lemoine et al. (2). In children with PFIC, biliary diversion can be curative or at least prolong the time to liver transplantation. Different surgical options for biliary diversion exist. Partial external biliary diversion (PEBD) depicts a procedure in which the gallbladder is either directly diverted as a stoma or with an interposition of a small bowel segment (Figure 1). Internal BD refers to the conversion of the bile from the gallbladder into the colon as a short cut to dramatically reduce enterohepatic circulation of bile salts. Another approach has been to bypass the terminal ileum as main location of bile reabsorption in terms of ileocaecal anastomosis, excluding approximately the last 100 cm of distal ileum. The latter two procedures, however, are believed to bring only temporary relief as other parts of small bowel adapt, and generally PEBD is favored. Consequently, in our cohort all children were subjected to PEBD except one child who received internal biliary diversion. This surgical approach is in line with others, such as Lemoine et al., who performed PEBD on all their cases (2). PEBD as well as internal drainage procedures can safely be carried out either laparoscopically or in an open fashion. Another option for surgical drainage is via a catheter into the gallbladder. Although less invasive, this method is often a poor option for extended time periods of biliary diversion due to the risk of infection and/or catheter dislocation. However, given the complexity of some of these children, it is a valuable option either for temporary partial biliary diversion in cases where surgery is not an option or in which a trial diversion is warranted. In our cohort, no child had a catheter-based drainage. If surgical options fail to reduce symptoms or the liver parenchyma changes towards fibrosis and cirrhosis, liver transplantation may become the only curative option.

Schukfeh et al., 2012 (5) showed that only 9 out of 24 patients needed liver transplantation after biliary diversion, for all PFIC subtypes. In our collective of 20 children with PFIC 2, only 7 had symptoms leading to biliary diversion. However, in most cases they did not improve clinically, and bile acids did not normalize. Four of these 7 children went on to have liver transplantation, and so did 6 of the children with PFIC 2 that did not have biliary diversion. Children with PFIC 3 in our collective did not receive biliary diversion but slowly developed liver fibrosis. Of these, 8 (72%) went on to liver transplant, which is in accordance to the literature (6).

In addition to changes in bile acid levels after biliary diversion (7) post interventional alteration in lipid metabolism. We equally addressed this in our collective. ATP8B1 (PFIC 1) is not only located on the canalicular membrane of hepatocytes but also acts as a stabilizer of the cell membrane against toxic substances in small bowel mucosa (8). This could lead to an increased lipid absorption in patients with PFIC 1. Our patients with PFIC 1 had higher levels of triglycerides than the other subtypes, which then normalized after biliary diversion supporting the hypothesis of Cielecka-Kuszyk et al. (7). Whether this alteration is of any clinical significance with regards to disease progression will need to be addressed in further studies.

Likewise, it is important to acknowledge the emergence of powerful pharmacological treatment options for PFIC. IBAT inhibitors have shown to effectively interrupt the enterohepatic circulation in patients with PFIC and to reduce both serum bile acids and pruritus (9–11). Whether they can reliably prolong NLS and avoid BD long term is an intriguing thought and remains to be evaluated.

Our study has limitations. First, our patient collective is small. Despite being a highly specialized superregional pediatric liver center, due to the rarity of the disease on average we only acquire roughly two new patients with PFIC per year, accumulating over 19 years to the 40 children presented in this study. Obviously, as with all rare diseases, and as was nicely shown in the recent paper by van Wessel et al., in depth insight will come especially from pooling patient data in large international, multi-center studies. However, while we continue to collaborate in multi-center studies, we find it important for each provider and center to additionally analyze and share data of larger individual cohorts, such is the case with our study. Another limitation in our study is the fact that we are not able to provide genetic distinction or description of sub-sub-types for children with PFIC 2 (for example, BSEP1, BSEP2, BSEP3) (Table 2). This is critical, since recent evidence suggests that there are certain subtypes within PFIC 2 that could potentially benefit from BD, and other that don't (3). We have now started to perform such sub-analysis for all subsequent new and future patients and encourage others who treat this disease to do so as well. Unfortunately, we could not accumulate this data retrospectively.

**Table 2:** Patient characteristics and genetics at baseline.

Case	Age at presentation (years)	Genetics	Reason for Presentation/Symptoms	PFIC	BD	LTx	Time from BD to LTx (month)
2	1	ATP8B1: p.K455N homozygous	For elective bile diversion surgery	1	1	1	93
4	13	n.a., clinical diagnosis	Evaluation for ltx	Low GGT PFIC, most likely type 1	0	0	
5	1	n.a., clinical diagnosis	Evaluation for ltx	Low GGT PFIC, most likely type 1	0	1	
7		ABCB4: p.T175A heterozygous	Itching	Low GGT PFIC, most likely type 1	0	0	
8	1	n.a., clinical diagnosis	Jaundice and itching	Low GGT PFIC, most likely type 1	1	0	
9	0	n.a., suspected Morbus Byler, clinical diagnosis	Bronchopneumonia	Low GGT PFIC, most likely type 1	1	0	
16		ATP8B1: c.2097 + 2T > C homozygous	Sister of a known Pt.	1	0	0	
17	0	ATP8B1: c.2097 + 2T > C homozygous	For further evaluation and therapy	1	1	1	28
24	1	n.a., clinical diagnosis	Itching	Low GGT PFIC, most likely type 1	1	0	
25	0	n.a., clinical diagnosis	Jaundice	Low GGT PFIC, most likely type 1	0	1	
6	2	ABCB11: p.E297G + p.G260D compound heterozygous	Elective biliary diversion	2	1	0	
10		n.a., clinical diagnosis	Not documented	Low GGT PFIC, most likely type 2	1	1	161
11	10	n.a., clinical diagnosis	Not documented	Low GGT PFIC, most likely type 2	1	0	
12	0	ABCB11: compound heterozygous	Cerebral hemorrhage	2	1	1	48
19	1	ABCB11: p.T1210P + p.N1173D compound heterozygous	Evaluation for ltx	2	0	1	
20		ABCB11: p.D496V + c.2178 + 1G > A compound heterozygous	Joint pain	2	1	0	
21	13	ABCB11: p.I213T + p.D482G compound heterozygous	Evaluation for ltx	2	1	1	17
26	6	ABCB11: p.S462R + p.I879R compound heterozygous	For evaluation of known PFIC	2	0	0	
27		ABCB11: p.S462R + p.I879R compound heterozygous	Not documented	2	0	1	
28	0	ABCB11: p.D473V heterozygous	For further evaluation and therapy	2	0	0	
29		ABCB11: p.I333F + p.V1164GfS*7 compound heterozygous	Cerebral hemorrhage	2	0	0	
30	1	ABCB11: p.T1210P homozygous	Evaluation for ltx	2	0	1	
31	0	ABCB11: c.77-1G > A + p.K461E compound heterozygous	Evaluation for ltx	2	0	1	
32	1	ABCB11: p.E297G homozygous	Evaluation for ltx	2	0	1	
35		ABCB11: p.W493* homozygous	Cholestasis syndrome	2	0	1	
36	6	ABCB11: c.2178 + 1G > C homozygous	Evaluation for ltx	2	1	1	80
37	1	ABCB11: p.D482G + p.T1210P compound heterozygous	Evaluation for ltx	2	0	1	
38	5	ATP8B1: p.N45T heterozygous, ABCB11: c.2178 + 1G > C homozygous	Evaluation for ltx	2	1	1	12
39	3	ABCB11: p.D482G + c.2178 + 1G > A compound heterozygous	Evaluation for ltx	2	0	1	
40	0	ABCB11: p.D482G homozygous	Evaluation for ltx	2	0	1	
1		ABCB4: p.P726l homozygous	Unclear hepatopathy with severe itching	3	0	1	
3		ABCB4: c.286 + 1G > A + p.Q1181E compound heterozygous	Evaluation in suspected cholestasis syndrome	3	0	0	
13		ABCB4: p.P726L homozygous	Not documented	3	0	1	
14		ABCB4: p.P726L homozygous	Family member of #13	3	0	1	
15	0	ABCB4: p.L23Hfs*16 + p.S27G compound heterozygous	Cirrhosis of the liver	3	0	1	
18	0	n.a., clinical diagnosis	Relocated to follow-up care close to home	High GGT PFIC, most likely type 3	0	0	
22	13	n.a., clinical diagnosis	Evaluation for ltx	High GGT PFIC, most likely type 3	0	1	
23		n.a., clinical diagnosis	Evaluation for ltx	High GGT PFIC, most likely type 3	0	1	
33		ABCB4: p.E528D heterozygous	Cholestasis syndrome	3	0	0	
34	0	ABCB4: p.P726L homozygous	Evaluation for family history positive for PFIC	3	0	1	

In conclusion, when evaluating a child for potential BD, it is important to consider that PFIC is a heterogenic disease with many different genetic subtypes that may respond differently to BD. In today's day and age, genetic analysis prior to BD should be considered mandatory. When considering BD, special attention should be directed towards children with PFIC 2 because BD may not be as successful when compared to PFIC 1, for example. Rather, in children with PFIC 2, further categorization according to genotypic severity (BSEP1, BSEP2, BSEP3) may be beneficial to predict the response to BD and NLS. In either case, pre- and postoperative BA levels should be closely followed, as a drop in bile acids following BD appear to correlate with NLS. Also, always it is important to continuously observe treatment outcomes with newly developed IBAT inhibitors, which will in evidently play into the decision-making process when evaluating children for possible surgical BD.

## Data Availability

The original contributions presented in the study are included in the article, further inquiries can be directed to the corresponding author/s.
